# Integrative Transcriptome Analysis of mRNA and miRNA in Pepper’s Response to *Phytophthora capsici* Infection

**DOI:** 10.3390/biology13030186

**Published:** 2024-03-14

**Authors:** Yuan Li, Nan Wang, Jianwen Guo, Xianjun Zhou, Xueyi Bai, Muhammad Azeem, Liyun Zhu, Lin Chen, Moli Chu, Hui Wang, Wei Cheng

**Affiliations:** 1 College of Life Sciences, Anhui Normal University, Wuhu 241000, China; 2 Anhui Provincial Key Laboratory of Molecular Enzymology and Mechanism of Major Metabolic Diseases, Anhui Normal University, Wuhu 241000, China; 3 Anhui Provincial Key Laboratory of the Conservation and Exploitation of Biological Resources, Anhui Normal University, Wuhu 241000, China; 4 College of Agriculture, Fujian Agriculture and Forestry University, Fuzhou 350002, China

**Keywords:** *Capsicum annuum*, *Phytophthora capsici*, transcriptome, miRNA, disease resistance

## Abstract

**Simple Summary:**

MicroRNAs (miRNAs) play important roles in the regulation of a plant’s response to a variety of pathogens. However, the regulatory networks of miRNAs and their targets in pepper’s response to *P. capsici* are largely unknown. Herein, integrative miRNA-seq and mRNA-seq analysis were performed to identify miRNAs and their target genes in pepper during *P. capsici* infection, and the results provide a valuable resource for better understanding the regulatory mechanisms between miRNAs and their targets in pepper’s defense against *P. capsici*.

**Abstract:**

Phytophthora blight of pepper is a notorious disease caused by the oomycete pathogen *Phytophthora capsici*, which poses a great threat to global pepper production. MicroRNA (miRNA) is a class of non-coding small RNAs that regulate gene expressions by altering the translation efficiency or stability of targeted mRNAs, which play important roles in the regulation of a plant’s response to pathogens. Herein, time-series mRNA-seq libraries and small RNA-seq libraries were constructed using pepper roots from the resistant line CM334 and the susceptible line EC01 inoculated with *P. capsici* at 0, 6, 24, and 48 h post-inoculation, respectively. For mRNA-seq analysis, a total of 2159 and 2971 differentially expressed genes (DEGs) were identified in CM334 and EC01, respectively. For miRNA-seq analysis, 491 pepper miRNAs were identified, including 330 known miRNAs and 161 novel miRNAs. Among them, 69 and 88 differentially expressed miRNAs (DEMs) were identified in CM334 and EC01, respectively. Examination of DEMs and their targets revealed 22 regulatory networks, predominantly featuring up-regulated miRNAs corresponding to down-regulated target genes. Notably, these DEM-DEG regulatory networks exhibited significant overlap between CM334 and EC01, suggesting that they might contribute to pepper’s basal defense against *P. capsici*. Furthermore, five selected DEMs (miR166, miR1171, miR395, miR530 and miRN2) and their target genes underwent qRT-PCR validation, confirming a consistent negative correlation in the expression patterns of miRNAs and their targets. This comprehensive analysis provides novel insights into the regulatory networks of miRNAs and their targets, offering valuable contributions to our understanding of pepper’s defense mechanisms against *P. capsici*.

## 1. Introduction

*Capsicum annuum*, which is commonly referred to as pepper, is one of the world’s most economically and agriculturally important vegetable and spice crops [1]. Phytophthora blight of pepper is a destructive disease caused by the oomycete pathogen *Phytophthora capsici*, which poses a great threat to the yield and quality of pepper production. All parts of the pepper plant including roots, stems, leaves and fruits can be infected, and symptoms can range from damping off and wilting to rotting. In addition to attacking solanaceous plants (e.g., pepper, tomato, and eggplant), *P. capsici* has a broad host range that can invades at least 26 plant families, including legume (snap and lima beans) and most cucurbit hosts, which poses great risks to food safety and security [2,3].

Although they morphologically resemble fungi, oomycetes such as *Phytophthora* spp. are phylogenetically related to diatoms and brown algae of the kingdom Stramenopila [4]. To date, natural resistance against *P. capsici* in pepper is inadequate. One of the pepper landraces ‘Criollo de Morelos 334’ (CM334) is the most well-known source of resistance to *P. capsici*. Although most of the commercial pepper cultivars released nowadays have a certain degree of resistance to *P. capsici*, a higher level of resistance has not been observed [1,5]. Once introduced to a field site, *P. capsici* proves challenging to control through chemical or cultural strategies, particularly under warm and wet environmental conditions [2]. In order to improve the measures for controlling this disease, it is essential to understand the molecular mechanisms in pepper defense against *P. capsici*.

MicroRNA (miRNA) is a class of non-coding small RNAs with length of about 18–24 bases. These miRNAs regulate gene expressions by altering translation efficiency or stability of targeted mRNAs, which play important roles in the regulation of a plant’s response to a variety of pathogens [6,7,8]. It has become clear that miRNAs are key components in transcriptional regulatory networks. To modulate expression of the genome, an individual miRNA may target more than one transcript and vice versa. Furthermore, some miRNAs have the capability to regulate target gene expression through histone modification or DNA methylation [7]. Recently, high-throughput sequencing technology has provided an effective way to identify and characterize the expression profiles of miRNAs in plant defense against pathogens. For instances, a total of 293 known miRNAs and 6 potential novel miRNAs were identified in *Arabidopsis thaliana* response to *P. capsici*, of which 33 miRNAs were differentially expressed during the infection [9]. In *Solanum lycopersicum*, 207 known miRNAs and 67 novel miRNAs were identified in tomato response to *P. infestans*, of which 70 miRNAs were manifested to change significantly when inoculated with the pathogen [10]. In *Glycine max*, 528 known and 555 novel miRNAs were identified in the soybean response to *P. sojae*, of which 74 known and 75 novel miRNAs were differentially expressed in the resistant line Nannong10-1; and 55 known and 43 novel miRNAs were differentially expressed in the susceptible line 06-070583 [11]. In *Capsicum annuum*, 79 known miRNAs and 477 novel miRNAs were identified in pepper’s response against *P. capsici* infection [12]. However, an integrative time-series transcriptome analysis of miRNA and mRNA in pepper’s defense against *P. capsici* at different infection stages has not been explored.

In this study, 24 mRNA-seq libraries and 24 small RNA-seq libraries were constructed using pepper roots from the resistant line CM334 and the susceptible line EC01 inoculated with *P. capsici* at 0, 6, 24, and 48 h post-inoculation (hpi), respectively. Combined with miRNA-seq and mRNA-seq analysis, the objective of this study was to reveal the regulatory networks of pepper miRNAs and their target genes in response to *P. capsici* infection, which may provide new insights into the molecular mechanisms of pepper defense against *P. capsici*.

## 2. Materials and Methods

### 2.1. Plant Materials and Growth Conditions

Based on phenotypic differences in disease resistance described previously [13], the *P. capsici*-resistant pepper landrace line ‘Criollo de Morelos 334’ (CM334) and the susceptible line ‘Early Calwonder 01’ (EC01) were used in this study. The pepper seeds were firstly sown in a soil mix and placed in the growth room for ~2 weeks under conditions as described previously [13]. Two weeks later, these pepper plants were washed and transferred to boxes containing Holland solution. Then, these soilless-cultivated pepper plants were grown for another ~2 weeks under the same condition described above prior to *P. capsici* inoculation [13].

### 2.2. Pathogen Culture and Inoculation Assay

The highly virulent *P. capsici* stain JX1 was cultured and induced zoospore production as described previously [14]. The density of *P. capsici* zoospores was adjusted to ~5 × 10^5^ zoospores/mL. Pepper roots were then immersed with the zoospore suspension and harvested at 0, 6, 24 and 48 h after *P. capsici* inoculation [13].

### 2.3. mRNA-Seq and miRNA-Seq Analysis

To obtain mRNA-seq and miRNA-seq data, the extracted RNA samples from pepper roots were used for mRNA-seq and small RNA-seq library construction and sequencing by Guhe Information Technology Co., Ltd. (Hangzhou, China).

For mRNA-seq, after filtering out the low-quality reads using FastQC (v0.12.0) and Trimmomatic softwares (v0.39) [15,16,17], clean reads were aligned to the CM334 genome version PEP (v1.55) (https://solgenomics.net/ftp/genomes/Capsicum_annuum/C.annuum_cvCM334/, accessed on 11 April 2022) using the HISAT2 program [18]. The number of mapped reads was normalized to fragments per kilobase million (FPKM). The differentially expressed genes (DEGs) were identified between the inoculated and mock-inoculated samples with a fold change >2 and FDR ≤ 0.01. Heat maps were constructed using the pheatmap (v1.0.12) package (https://cran.r-project.org/web/packages/pheatmap/, accessed on 11 April 2022). The Z-scores of the miRNA-seq data were analyzed using the ggplot2 (v3.4.2) package (https://cran.r-project.org/web/packages/ggplot2/, accessed on 11 April 2022). The Kyoto Encyclopedia of Genes and Genomes (KEGG) pathway enrichment analysis was performed using the KOBAS 3.0 web-based toolkit [19].

For miRNA-seq, after filtering out the low-quality reads by FastQC and Cutadapt (v4.4) softwares [20], the high-quality clean reads with a length of 18–30 nt were then aligned to the latest published pepper miRNA database (http://plantsrnas.org/miRNAlist.jsp?species=Capsicum_annuum, accessed on 11 April 2022) using Bowtie2 (v2.5.1) software [21]. Novel miRNAs were also predicted using miRDP2 (v1.1.4) [22]. The expression level of miRNAs was normalized to transcripts per million (TPM). Differentially expressed miRNAs [DEMs, with padj < 0.05, and log2 (fold change) > 1 or <−1] between the inoculated and mock-inoculated samples were identified using DEseq2 (v1.42.1) software. Prediction of miRNA target genes was performed using psRNATarget (2017 release) and psRobot (v1.2) software [23,24], and the intersection of these predicted results were obtained using the online Wayne network (https://bioinfogp.cnb.csic.es/tools/venny/index.html, accessed on 11 April 2022). Then, we calculated the Pearson correlation coefficient (PCC) between DEMs and their target genes with a corr < −0.2, and plotted the miRNA–target genes network using Cytoscape (3.9.1) software [25].

### 2.4. RNA Extraction and Quantitative Real-Time PCR (qRT-PCR) Analysis

RNA samples were extracted from *P. capsici*-inoculated pepper roots as described previously [14]. The first-strand cDNA was reversely transcribed using the MonScript™ miRNA First Strand cDNA Synthesis Kit (Tailing Reaction) or MonScript™ RTIII Super Mix with dsDNase (Two-Step) according to the manufacturer’s instructions. To determine the expression profiles of selected miRNAs and their target genes, quantitative real-time PCR was performed using specific primers (Supplementary Materials: Table S1) using the MonAmp™ ChemoHS qPCR Mix. Each treatment was performed with three independent biological replicates. We analyzed the data using the Livak method, and calculated the normalized relative expression level (2^−ΔΔCT^) [26].

## 3. Results

### 3.1. High-Throughput mRNA Sequencing and Differentially Expressed Gene (DEG) Analysis

To investigate defense mechanisms of pepper against *P. capsici*, time-series mRNA-seq data from the susceptible pepper line EC01 and the resistant pepper line CM334 at 0, 6, 24 and 48 hpi were analyzed. In total, 24 samples (2 genotypes × 4 time points × 3 biological replicates) were obtained for mRNA-seq library construction. After filtering out the low-quality reads, approximately 81.1 and 83.0 million clean reads were obtained in each sample of CM334 and EC01, respectively (Supplementary Materials: Table S2).

**Figure 1:**
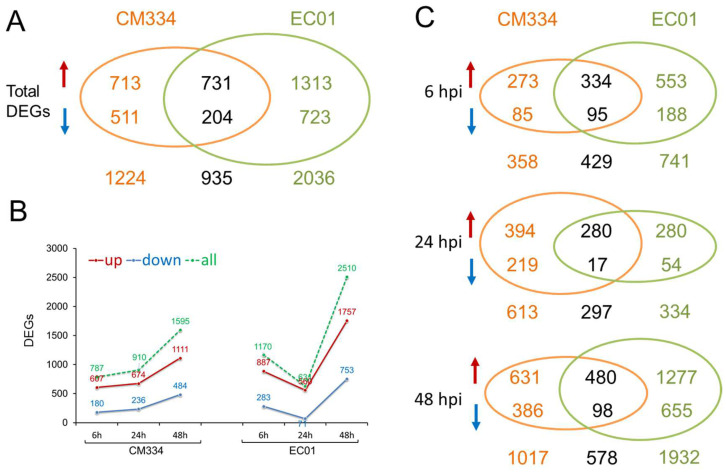
Differentially expressed genes (DEGs) identified in the pepper lines CM334 and EC01 against *P. capsici* infection. (**A**) In total, 2159 and 2971 DEGs were identified in CM334 and EC01 during *P. capsici* infection, respectively, and 935 DEGs overlapped between CM334 and EC01. (**B**) DEGs were identified in CM334 and EC01 at 6, 24 and 48 h after *P. capsici* inoculation, respectively. (**C**) Venn diagrams of DEGs identified from CM334 and EC01 at each time point after *P. capsici* inoculation, respectively. The upward arrows represent the significantly up-regulated genes, and the downward arrows represent the significantly down-regulated genes.

**Figure 2:**
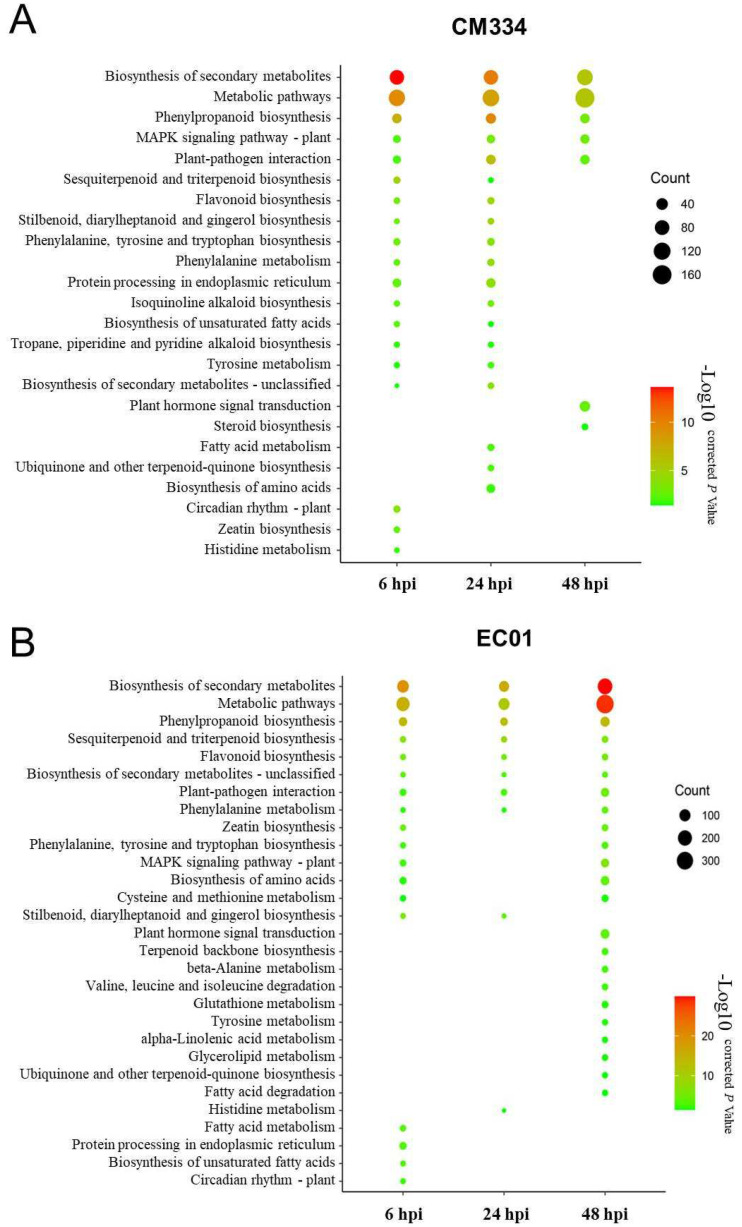
Kyoto Encyclopedia of Genes and Genomes (KEGG) pathway enrichment analysis of differentially expressed genes (DEGs) in pepper lines CM334 and EC01 against *P. capsici* infection. (**A**) KEGG pathway enrichment analysis of DEGs in CM334 at various time stages after *P. capsici* infection. (**B**) KEGG pathway enrichment analysis of DEGs in EC01 at the indicated time points after *P. capsici* infection. The size of the dot indicates the number of DEGs in the corresponding pathway.

Following alignment to the pepper CM334 genome, we normalized the number of mapped reads to FPKM and identified differentially expressed genes (DEGs) between the inoculated and mock-inoculated samples. Compared with 0 hpi, a total of 2159 DEGs with 1444 up-regulated and 715 down-regulated genes were identified in the resistant pepper line CM334. In comparison, 2971 DEGs with 2044 up-regulated and 927 down-regulated genes were identified in the susceptible pepper line EC01. These results indicated that it can trigger extensive transcriptional reprogramming in both lines during the *P. capsici* infection, with more DEGs in EC01 (Figure 1A and Figure S1). Interestingly, time-resolved transcriptome analysis indicated that there are more DEGs in EC01 at 6 hpi and 48 hpi, while there are more DEGs in CM334 at 24 hpi (Figure 1B,C). KEGG enrichment analysis of these identified DEGs showed that the terms of metabolic pathways, especially secondary metabolite biosynthesis (e.g., phenylpropanoid biosynthesis), are most significantly enriched among all these comparison combinations (Figure 2).

### 3.2. High-Throughput Small RNA Sequencing and Differentially Expressed miRNA (DEM) Analysis

To identify pepper miRNAs in response to *P. capsici* inoculation, time-series small RNA-seq data from the susceptible line EC01 and the resistant line CM334 at 0, 6, 24 and 48 hpi were analyzed. Similarly, a total of 24 samples (2 genotypes × 4 time points × 3 biological replicates) were obtained for small RNA-seq library construction. After filtering out the low-quality reads of the small RNA-seq data (Supplementary Materials: Table S3), clean reads with length of 18–30 nt were aligned to the latest published pepper miRNA database, and predicted novel miRNAs using the miRDP2 (v1.1.4) software [22].

**Figure 3:**
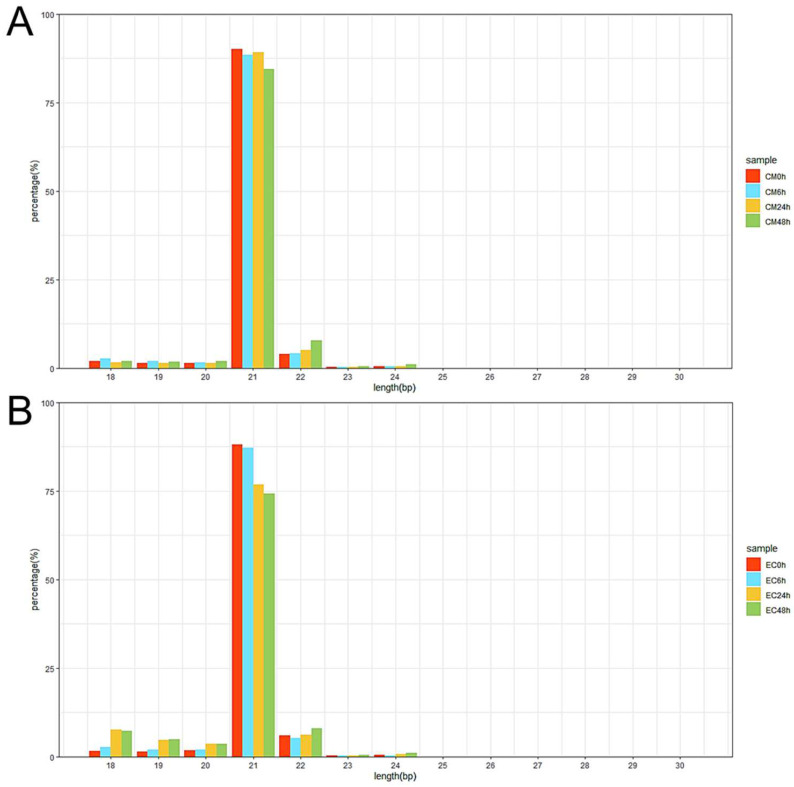
Length distribution of miRNAs in pepper at different time points after *P. capsici* inoculation. (**A**) Length distribution of miRNAs was obtained from CM334 at 0, 6, 24 and 48 h after *P. capsici* inoculation. (**B**) Length distribution of miRNAs was obtained from EC01 at the indicated time points after *P. capsici* inoculation. The *x*-axis shows the miRNA sequence sizes from 18 to 30 nt; the *y*-axis shows the percentage of miRNA for every given size.

**Figure 4:**
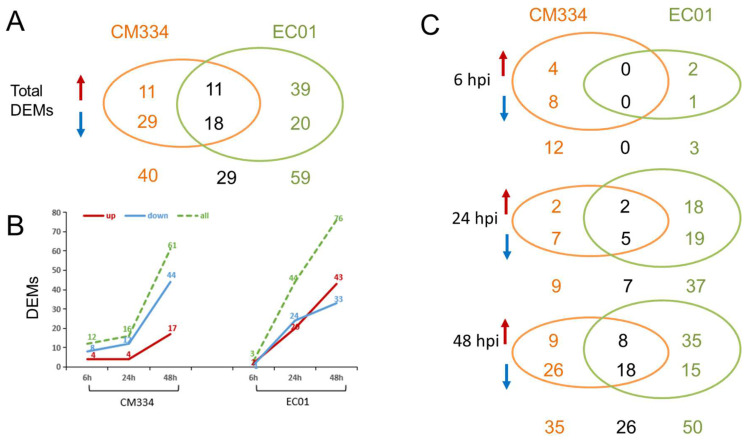
Differentially expressed miRNAs (DEMs) identified in the pepper lines CM334 and EC01 against *P. capsici* infection. (**A**) In total, 69 and 88 DEMs were identified in CM334 and EC01 during the *P. capsici* infection, respectively, and 29 DEMs overlapped in both lines. (**B**) DEMs were identified in CM334 and EC01 at 6, 24 and 48 h after *P. capsici* inoculation, respectively. (**C**) Venn diagrams of DEMs identified from CM334 and EC01 at each time point after *P. capsici* inoculation, respectively. The upward arrows represent the significantly up-regulated miRNAs, and the downward arrows represent the significantly down-regulated miRNAs.

**Figure 5:**
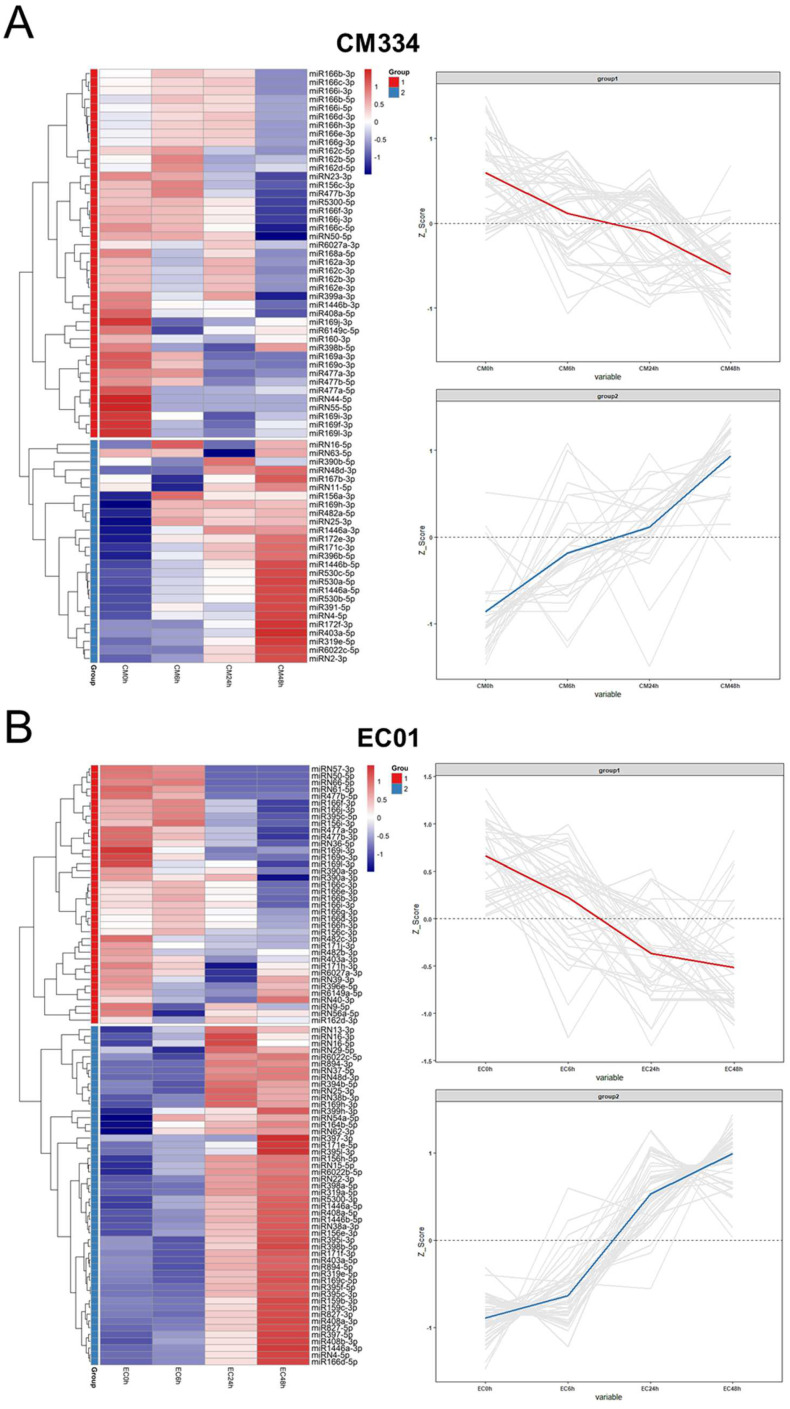
Expression profiles of differentially expressed miRNAs (DEMs) in the pepper lines CM334 and EC01 against *P. capsici* infection. (**A**) The transcriptional patterns of DEMs in CM334 at 0, 6, 24 and 48 h after *P. capsici* inoculation. (**B**) The transcriptional patterns of DEMs in EC01 at the indicated time points inoculated with *P. capsici*. The heat maps (left panel) of DEMs were constructed using the pheatmap package. The Z-scores of miRNA-seq data (right panel) were used for analysis of DEM expression trends using the ggplot2 package.

A total of 330 known miRNAs and 161 novel miRNAs were identified, and most of these pepper miRNAs were 21 nt in length (Figure 3). Compared with mock-inoculated samples (0 hpi), 69 and 88 differentially expressed miRNAs (DEMs) were identified in CM334 and EC01, respectively. Among them, 29 DEMs were commonly identified in both pepper lines (Figure 4A). At 6 hpi, 12 DEMs and 3 DEMs were identified in CM334 and EC01, respectively. At this time point, the number of up- and down-regulated DEMs in CM334 was greater than that in EC01, which may suggest that some miRNAs in CM334 responded earlier to *P. capsici* infection. At 24 hpi, 16 DEMs were identified in CM334 and 44 DEMs were identified in EC01; the number of DEMs in CM334 was far less than that in EC01, indicating the difference of miRNA-mediated defense responses between the resistant and susceptible pepper lines. At 48 hpi, 61 DEMs and 76 DEMs were identified in CM334 and EC01, respectively, suggesting that a large amount of pepper miRNAs are involved in the response to *P. capsici* at this infection stage (Figure 4B,C and Figure S2). Based on the expression profiles of DEMs identified in CM334 and EC01, it was found that most of these DEMs show trends of continuous up-regulation or down-regulation during the infection. In CM334, the number of up-regulated DEMs (26) was less than that of down-regulated DEMs (43); while in EC01, the number of up-regulated DEMs (50) was more than that of down-regulated DEMs (38) during the *P. capsici* infection (Figure 5).

### 3.3. Combined Analysis of miRNA and mRNA Transcriptome

**Figure 6:**
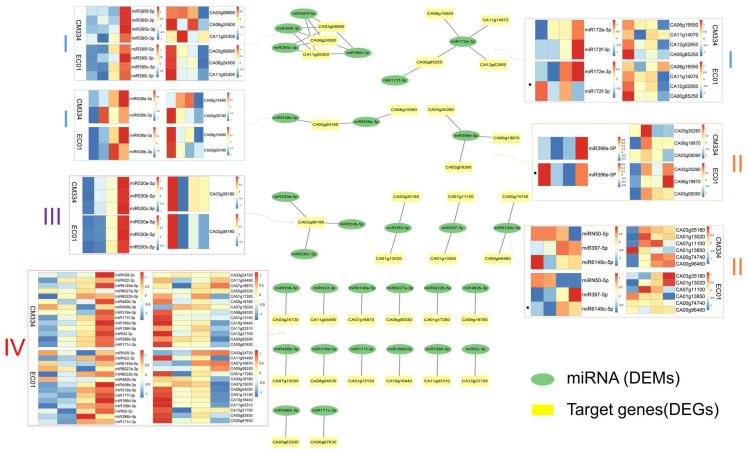
Regulatory networks of differentially expressed miRNAs (DEMs) and their differentially expressed target genes (DEGs) in pepper lines CM334 and EC01 against *P. capsici* infection. These 22 DEM-DEG regulatory networks can be divided into four groups. Samples in the heat maps from left to right are 0 hpi, 6 hpi, 24 hpi and 48hpi. Group I consists of DEM-DEG networks of multiple DEGs targeted by multiple DEMs, group II consists of multiple DEGs targeted by single DEMs, group III consists of multiple DEMs targeting single DEGs, and group IV consists of single DEMs targeting single DEGs.

We performed target gene prediction for all miRNAs obtained by miRNA-seq in this study, and the results showed that a total of 424 miRNAs were predicted to target 1725 genes. Moreover, in CM334, 62 DEMs were predicted to target 262 genes, forming 347 miRNA–target gene pairs. In EC01, 79 DEMs were predicted to target 436 genes, forming 536 miRNA–target gene pairs. To further identify target DEGs that were negatively correlated with DEMs, we screened 22 DEM-DEG regulatory networks and found that most of them were up-regulated miRNAs corresponding to down-regulated target genes. Moreover, these DEMs-DEG regulatory networks greatly overlapped between CM334 and EC01, suggesting that they might contribute to basal defense against *P. capsici* (Figure 6).

These DEM-DEG regulatory networks could be classified into four groups: group I, the regulatory networks of multiple target DEGs regulated by multiple DEMs: (1) the miR395 family has four miRNAs (miR395f-5p, miR395c-3p, miR395l-3p, and miR395i-3p), targeting three genes (CA03g06690, sulfate adenylyltransferase; CA11g05300, sulfate/bicarbonate/oxalate exchanger and transporter sat-1; CA06g24500, usaric acid resistance protein). During the *P. capsici* infection, these four miRNAs were up-regulated in both CM334 and EC01, and their target genes were down-regulated in both lines; (2) the miR172 family has two miRNAs (miR172e-3p, miR172f-3p) that target four genes. MiR172e-3p was up-regulated in both CM334 and EC01, while miR172f-3p was significantly up-regulated in CM334 and showed down-regulation in EC01 during the infection. Their target genes CA06g19550 (BAH domain-containing protein), CA11g14070 (AP2 transcription factor), CA00g85250 (AP2 transcription factor) and CA12g02850 (unknown function protein) were significantly decreased in the both lines; (3) there are two miRNAs (miRN38a-3p and miRN38b-3p) in the miRN38 family that target two genes (CA08g15490 and CA05g09180). MiRN38a-3p and miRN38b-3p are up-regulated in both lines after *P. capsici* infection. The target gene CA08g15490 (LEXYL1 protein) is significantly down-regulated in EC01, while it was increased at 6 and 24 hpi, then decreased at 48 hpi in CM334. The other target gene CA05g09180 (JHL10I11.7 protein) was down-regulated in both lines (Figure 6).

For group II, the regulatory networks of multiple target DEGs were regulated by one DEM: (1) miR396e-5p targets three genes (CA03g30260, CA06g19870, CA03g08260). The miR396e-5p was significantly up-regulated in CM334, but down-regulated in EC01 during *P. capsici* infection. Correspondingly, its target genes CA03g30260 (heat shock protein), CA06g19870 (protein binding protein) and CA03g08260 (bHLH120-like transcription factor) were down-regulated in CM334 and up-regulated in EC01; (2) miRN50-5p, miR397-5 and miR6149c-5p corresponded to two target genes, respectively. Among them, miRN50-5p was down-regulated in CM334 and EC01, and its target genes CA03g35180 (Lipoxygenase) and CA01g13020 (F-box family protein) were up-regulated in the two lines during the infection. MiR397-5p was up-regulated both in CM334 and EC01, and its target gene CA01g13850 (E3 ubiquitin-protein ligase) was down-regulated in both lines; while its other target gene CA07g11100 (Laccase 110a) increased at 6 and 24 hpi, then decreased at 48 hpi in both lines. The miR6149c-5p was down-regulated in CM334, while up-regulated in EC01 after *P. capsici* infection. Its target genes (CA00g74740, F-box family protein; CA00g96460, Ubiquitin-protein ligase) were up-regulated in CM334, but showed no significant changes in EC01 (Figure 6).

For group III, the regulatory networks of multiple DEMs collectively regulated one target DEG: the miR530 family has three miRNAs (miR530a-5p, miR530b-5p and miR530c-5p), regulating one target gene CA03g06190 (unknown function protein). These three miRNAs were up-regulated in both CM334 and EC01, and their target gene CA03g06190 was down-regulated in the two lines during *P. capsici* infection (Figure 6).

For group IV, the regulatory networks of one target DEG were regulated by one DEM: for instance, miR6022b-5p, miR171f-3p, miR166d-5p, miR156h-5p, miR396b-5p, miR171c-3p, miRN2-3p and MiRN22-3p were all up-regulated in both CM334 and EC01 after *P. capsici* inoculation, and their corresponding target genes CA01g17280 (aspartokinase-homoserine dehydrogenase), CA01g13150 (GRAS family transcription factor), CA10g16440 (serine/threonine protein kinase), CA11g03310 (LIGULELESS1 protein), CA00g82930 (transcription factor), CA00g67630 (GRAS family transcription factor), CA12g21700 (receptor-like protein 12) and CA11g04490 (cysteine desulfurase) were down-regulated in both lines, respectively (Figure 6).

### 3.4. Expression Profiles of miRNAs and Their Target Genes Examined by qRT-PCR Validation

**Figure 7:**
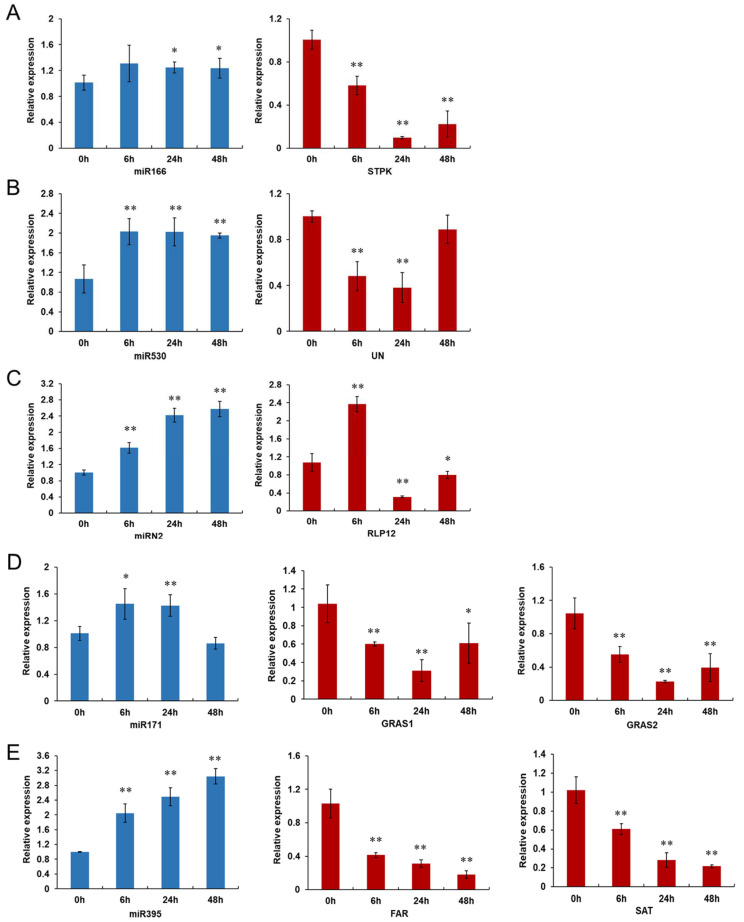
The qRT-PCR verification of five miRNAs and their target genes in pepper’s response against *P. capsici* infection. (**A**) miR166, (**B**) miR530, (**C**) miRN2, (**D**) miR171 and (**E**) miR395 and their target genes were selected for analyzing their expression patterns by qRT-PCR. STPK (serine/threonine protein kinase, CA10g16440); UN (unknown function protein, CA03g06190); RLP12 (receptor-like protein 12, CA12g21700); GRAS1 (GRAS family transcription factor, CA00g67630); GRAS2 (GRAS family transcription factor, CA01g13150); FAR (fusaric acid resistance protein, CA06g24500); SAT (sulfate adenylyltransferase, CA03g06690). Samples were collected from pepper line CM334 at 0, 6, 24 and 48 h after *P. capsici* inoculation. Each treatment was performed with three independent biological replicates. Asterisks indicate statistically significant differences as compared with mock-inoculated samples (0 hpi) using the least significant difference (LSD) test (* *p* < 0.05; ** *p* < 0.01).

To validate the reliability of the transcriptome sequencing data, five miRNAs (four known and one novel miRNA) and their corresponding target genes were selected for analyzing their expression patterns in CM334 by qRT-PCR. As shown in Figure 7, these five miRNAs (miR166, miR530, miRN2, miR171 and miR395) were up-regulated after *P. capsici* inoculation, and their corresponding target genes were down-regulated during the infection, except for three miRNA–gene pairs at specific time points. More specifically, miR166 was up-regulated and its target gene CA10g16440 (serine/threonine protein kinase) was correspondingly down-regulated during the infection. Similarly, miR395 showed up-regulation upon *P. capsici* inoculation, and its target genes CA06g24500 (fusaric acid resistance protein) and CA03g06690 (sulfate adenylyltransferase) were observed with a decreased expression level during the infection. miR530 exhibited consistent up-regulation throughout all the infection time points. Its target gene CA03g06190 (unknown function protein) was significantly down-regulated at 6 and 24 hpi, but did not significantly change at 48 hpi. miR2 was up-regulated during all the infection time points. The expression level of its target gene CA12g21700 (receptor-like protein 12) was significantly decreased at 24 and 48 hpi, with significant up-regulation at 6 hpi. miR171 was up-regulated at 6 and 24 hpi, while it showed no significant change at 48 hpi. Its target genes CA00g67630 (GRAS family transcription factor) and CA01g13150 (GRAS family transcription factor) were significantly decreased during all the infection time points. Thus, it was shown that there was an almost negative correlation across the expression patterns between these miRNAs and their target genes, which were similar to the results from the high-throughput sequencing.

## 4. Discussion

Plant defenses against pathogens are largely regulated by complicated transcriptional and post-transcriptional networks, and miRNAs might be important modulators during infection. To date, several miRNAs from different plant species have been documented to participate in plant defense against a variety of *Phytophthora* spp. [27,28,29,30,31,32,33,34,35,36,37,38]. Although phytophthora blight disease frequently causes heavy losses to global pepper production, our knowledge on the regulatory networks of miRNAs and their target genes in pepper’s defense against *P. capsici* is still limited.

In the present study, time-series mRNA-seq libraries and small RNA-seq libraries were constructed using pepper roots from the resistant line CM334 and the susceptible line EC01 inoculated with *P. capsici* at 0, 6, 24, and 48 hpi. For mRNA-seq analysis, a total of 2159 and 2971 DEGs were identified in CM334 and EC01, respectively (Figure 1). And it was revealed that the terms related to cellular metabolic pathways, especially phenylpropanoid biosynthesis, are significantly enriched during the infection (Figure 2). Similar results had been reported in a recent study using RNA-seq analysis from pepper roots of the resistant line CM334 and the susceptible line NMCA10399 after *P. capsici* infection [39], which indicated that the phenylpropanoid pathways might play critical roles in pepper’s resistance to *P. capsici*. For example, monolignols are synthesized from phenylalanine via the phenylpropanoid pathway. Plant laccases catalyze the last step of monolignol oxidation and polymerization in the canonical lignin biosynthesis pathway [40]. In this study, a plant laccase gene CA07g11100, which targeted by miR397, was up-regulated at 3 and 24 hpi, then decreased at 48hpi in both pepper lines (Figure 6). In addition to phenylpropanoid pathways, our findings revealed that sulfate adenylyltransferase (CA03g06690) and fusaric acid resistance protein (CA06g24500), which were targeted by miR395, were down-regulated during the infection, indicating their involvement in pepper and *P. capsici* interactions (Figure 7).

In *Capsicum annuum*, a previous study identified 79 known miRNAs and 477 novel miRNAs from control and *P. capsici*-inoculated leaves of resistant and susceptible pepper genotypes. Among them, 29 known and 157 novel miRNAs in the resistant genotype and 30 known and 177 novel miRNAs in the susceptible genotype revealed differential expression patterns during *P. capsici* infection [12]. In this study, the comprehensive miRNA-Seq analysis of pepper roots from CM334 and EC01 against *P. capsici* resulted in the identification of a diverse set of pepper miRNAs. Notably, among these 491 identified miRNAs, 330 were known miRNAs, while 161 were characterized as novel miRNAs. The observed variations in miRNA expression patterns are indicative of the dynamic regulatory responses within the pepper plants during the different infection stages. Herein, 69 and 88 DEMs were identified in CM334 and EC01, respectively, which highlights the distinct regulatory landscapes in these two pepper lines against *P. capsici* (Figure 4). These findings underscored the complexity of miRNA-mediated response to *P. capsici*, contributing valuable insights into the nuanced interactions between the pathogen and different pepper genotypes.

Upon analyzing the expression profiles of these identified DEMs in CM334 and EC01, distinct trends emerged, revealing a consistent pattern of continuous up-regulation or down-regulation in both lines throughout the infection stages. In CM334, a notable observation was the prevalence of fewer up-regulated DEMs compared to down-regulated DEMs. Conversely, in EC01, the pattern was characterized by a higher number of up-regulated DEMs than down-regulated DEMs (Figure 5). The differential trends observed in CM334 and EC01 suggest genotype-specific responses to *P. capsici*, emphasizing the importance of considering the diverse regulatory mechanisms playing important roles in different pepper lines during infection.

To explore the potential regulatory relationships between DEMs and their target genes, we conducted an analysis to identify 22 DEM-DEG regulatory networks. Notably, a predominant observation was the prevalence of up-regulated miRNAs corresponding to down-regulated target genes within these networks. This suggests a regulatory mechanism where the up-regulation of specific miRNAs correlates with the down-regulation of their target genes, indicating potential post-transcriptional regulation in response to *P. capsici* infection (Figure 6). Although some DEMs (e.g., miR172f-3p, miR396e-5p, miR6149c-5p) and their target genes revealed different expression patterns in the resistant and susceptible genotypes, the significant overlap observed in these DEM-DEG regulatory networks between CM334 and EC01 suggests a shared regulatory response, hinting at a potential contribution to pepper’s basal defense against *P. capsici*. The consistency in these regulatory networks across different pepper lines underscores the importance of these identified miRNA–target gene interactions in shaping the overall defense mechanisms against the pathogen.

Furthermore, we selected five DEMs (miR166, miR1171, miR395, miR530 and miRN2) along with their predicted target genes for qRT-PCR analysis (Figure 7). This experimental approach aimed to confirm and strengthen the observed expression patterns of these miRNAs and their respective targets identified from our high-throughput sequencing. To our knowledge, there is no previous report documenting the roles of these miRNAs in pepper defence to *P. capsici*; however, their functional roles in other plant species against pathogen infection has been reported. In *Solanum lycopersicum*, miR166 was differentially regulated upon ToLCNDV infection in two contrasting tomato cultivars, and it could target and suppress the expression of a negative regulator (SlyHB) in plant defense, which encodes a class III homeodomain-leucine zipper transcription factor [41]. In rice, miR395 could target and suppress the expression of an ATP sulfurylase gene (OsAPS1) and two sulfate transporter genes (OsSULTR2;1 and OsSULTR2;2) to promote sulfate accumulation, resulting in broad-spectrum resistance to bacterial pathogens *Xanthomonas oryzae* pv. *oryzae* and *X. oryzae* pv. *oryzicola* in miR395-overexpressing plants [42]. Overexpression of miR530 could compromise blast disease resistance to fungal pathogen *Magnaporthe oryzae* and lead to reduced grain yields and late maturity, while blocking miR530 using a target mimic (MIM530) leads to enhanced resistance, increased grain yields and early maturity [43]. In this study, we revealed that there is an almost negative correlation across the expression patterns between these five selected miRNAs and their putative target genes, except for three miRNA–gene pairs at specific time points (miR530 and its target gene at 48 hpi, miR2 and its target gene at 6 hpi, miR171 and its target gene at 48 hpi). It is noted that the transcriptional regulatory networks of some specific genes are more complex than simple one-to-one relations. Indeed, these targeted genes are not only regulated by their corresponding miRNAs, but also might be modulated by some other regulation factors such as transcription activators/repressors, long non-coding RNAs (lncRNA), circular RNA (circRNA), micropeptides (miPs), etc. [7,44]. To elucidate the underlying mechanism of plant immunity mediated by these DEMs-DEG regulatory networks, further investigations to confirm and characterize their possible roles of specific miRNAs and their targets in pepper defense to *P. capsici* infection are required.

## 5. Conclusions

In conclusion, combined with miRNA-seq and mRNA-seq analysis, this study delved into the elaborate dynamics of pepper’s response against *P. capsici*, unraveling distinct regulatory landscapes during infection stages between the resistant line CM334 and the susceptible line EC01. Herein, a total of 491 pepper miRNAs were identified, including 330 known miRNAs and 161 novel miRNAs. Among them, 69 and 88 differentially expressed miRNAs (DEMs) were identified in CM334 and EC01, respectively. Moreover, 2159 and 2971 differentially expressed genes (DEGs) were identified in CM334 and EC01, respectively, indicating extensive transcriptional reprogramming in response to *P. capsici*, with a higher number of DEMs and DEGs observed in EC01. These identified pepper miRNAs, including known and novel ones, underscored the complexity of miRNA-mediated regulation in pepper plants facing this notorious pathogen. Examining the expression profiles of DEMs in CM334 and EC01, we observed consistent trends of up-regulation or down-regulation throughout the infection stages. Notably, the distinct patterns between the lines, with fewer up-regulated DEMs in CM334 and more in EC01, highlighted the genotype-specific responses. Our exploration of DEM-DEG regulatory networks revealed a prevalent correlation between up-regulated miRNAs and down-regulated target genes, suggesting a potential post-transcriptional regulatory mechanism in response to *P. capsici* infection. The substantial overlap in these networks between CM334 and EC01 suggested a shared regulatory response contributing to pepper’s basal defense against *P. capsici*. Furthermore, our experimental validation of selected DEMs and their target genes through qRT-PCR added empirical support to our computational findings, enhancing the reliability of these identified miRNA–target gene relationships. Our newfound knowledge of miRNAs and target genes may offer a promising avenue for tailored approaches to mitigate phytophthora blight in pepper crops.

## Data Availability

The data presented in this study are available in the article. Further information is available upon request from the corresponding authors.

## Supplementary Materials

The following supporting information can be downloaded at: https://www.mdpi.com/article/10.3390/biology13030186/s1. Table S1: Primers used in this study; Table S2: mRNA-seq libraries of pepper samples infected with *P. capsici*; Table S3: Small RNA-seq libraries of pepper samples infected with *P. capsici*; Figure S1: Volcano plots of differentially expressed genes (DEGs) in pepper’s response to *P. capsici* infection; Figure S2: Volcano plots of differentially expressed miRNAs (DEMs) in pepper’s response to *P. capsici* infection.
