# Supplementary material for: Integrative Transcriptome Analysis of mRNA and miRNA in Pepper’s Response to Phytophthora capsici Infection
Source: Biology (Basel). 2024 Mar 14;13(3):186. doi: 10.3390/biology13030186 (PMC10967991; doi:10.3390/biology13030186)
Supplement: Supplementary file 1 [file biology-13-00186-s001.zip › biology-2871354-supplementary.pdf]

Table S1 Primers used in this study.

Gene	Forward primers (5'→3')	Reverse primers(5'→3')
Primers used for qRT-PCR verification of miRNAs' expression patterns		
STPK	GGTTGGGAACAGAGACAGGA	CTCCAGGTACTTGAGGTGGA
UN	TTTGCGCTTGAGACTAGGGA	GGAGACATACTGGCTTCCCA
RLP12	TCGTGGAGATACATATTACAAAGAT	TCAGCACATGAAGTGCAATGA
GRAS1	TTCAACGTCATGGTCACTGC	CGGTCACAACCTCTGTCAAC
GRAS2	CCAGGCCGAATGTGTTGTTA	TCCAAGTAGCAGCTGACAGG
FAR	AGGTATGCAAGTGAAGAGGAGA	CCTTCACAAGTCCATCCATGC
SAT	AAGTTACCATCATTATCAAGGG	CTTCAATAGGAATGTGTACCAA
CaActin	AGGGATGGGTCAAAAGGATGC	GAGACAACACCGCCTGAATAGC
Primers used for qRT-PCR verification of target genes' expression patterns		
miR166	GCGCGAGAATGTTGTCTGGT	Universal-qPCR 3' primer
miR530	GCGTGCATTTGCACCTGC	Universal-qPCR 3' primer
miRN2	CGCGTGGTATTGTTCCGTT	Universal-qPCR 3' primer
miR171	CGTGATTGAGCCGTGCC	Universal-qPCR 3' primer
miR395	GCGCTGAAGTGTTTGGGG	Universal-qPCR 3' primer
U6	GATTTGTGCGTGTCATCCTT	GGGGACATCCGATAAAATTGG

Table S2 mRNA-seq libraries of pepper samples infected with *P. capsici*

Sample	Clean reads	Clean bases (G)	Q20(%)	Q30(%)	GC(%)
CM0h-1	78188400	10.76	98.74	95.11	44.74
CM0h-2	83988478	11.47	98.54	94.52	43.83
CM0h-3	77619568	10.57	98.53	94.49	43.48
CM6h-1	90176120	12.39	98.25	94.25	43.23
CM6h-2	72146742	9.92	98.65	94.88	43.79
CM6h-3	86090580	11.83	98.64	94.84	43.47
CM24h-1	72686490	9.41	97.43	92.58	42.97
CM24h-2	73167344	9.76	97.87	93.52	43.42
CM24h-3	102243124	13.63	97.96	93.63	43.39
CM48h-1	96355956	12.91	98.14	94.05	43.00
CM48h-2	74280766	9.92	97.99	93.68	43.19
CM48h-3	66453164	9.00	97.96	93.90	43.27
EC0h-1	76148726	10.41	98.62	94.78	43.81
EC0h-2	73300914	9.94	98.38	94.07	43.46
EC0h-3	87320894	11.89	98.50	94.40	43.56
EC6h-1	91135202	12.47	98.60	94.72	43.67
EC6h-2	72283878	10.05	98.88	95.40	43.13
EC6h-3	68945514	9.61	98.94	95.57	43.72
EC24h-1	102039510	14.53	97.90	94.15	42.97
EC24h-2	102660800	13.62	97.77	93.14	42.65
EC24h-3	106700378	14.18	98.00	93.64	43.08
EC48h-1	66831516	9.00	96.28	89.70	42.95
EC48h-2	76507054	10.01	97.59	92.72	42.92
EC48h-3	72367592	9.76	98.02	93.84	42.65

Note: CM represents the resistant pepper line CM334, EC represents the susceptible pepper line EC01; 0 h, 6 h, 24 h and 48 h represent the four inoculation time points; 1, 2 and 3 represent three independent biological replicates.

Table S3 Small RNA-seq libraries of pepper samples infected with *P. capsici*

Sample	Clean reads	Clean bases	Q20	Q30	GC
CM0h-1	12146806	276515956	0.995471	0.985914	0.497295
CM0h-2	14682624	338146850	0.995796	0.987363	0.482447
CM0h-3	11764775	261805216	0.99536	0.984992	0.498851
CM6h-1	11584353	262161712	0.99545	0.985842	0.521355
CM6h-2	10789385	249561823	0.994554	0.98287	0.527683
CM6h-3	11144817	256795311	0.995563	0.986643	0.522855
CM24h-1	12381027	274606996	0.995651	0.986476	0.508253
CM24h-2	13863230	325028602	0.99574	0.986642	0.502204
CM24h-3	14126060	338536648	0.995427	0.985786	0.507608
CM48h-1	11969219	282999989	0.995181	0.984612	0.49504
CM48h-2	13450691	314046266	0.995024	0.984261	0.500074
CM48h-3	11623623	274008898	0.994303	0.982685	0.492085
EC0h-1	12175021	285054384	0.995307	0.98512	0.488715
EC0h-2	10216739	229064792	0.995221	0.985036	0.496961
EC0h-3	9972533	218831100	0.995129	0.984569	0.514259
EC6h-1	7766172	170519399	0.994927	0.984413	0.522695
EC6h-2	9662260	217640236	0.995273	0.985149	0.500806
EC6h-3	8442852	183894064	0.994417	0.994417	0.53662
EC24h-1	5978561	131309431	0.995478	0.985886	0.530819
EC24h-2	5486461	116961471	0.995171	0.985107	0.539875
EC24h-3	5015176	106585619	0.995464	0.995464	0.538274
EC48h-1	9171606	203779988	0.995314	0.985435	0.526667
EC48h-2	8195853	186716518	0.995526	0.985938	0.53002
EC48h-3	6845811	157969089	0.995376	0.98551	0.526416

Note: CM represents the resistant pepper line CM334, EC represents the susceptible pepper line EC01; 0 h, 6 h, 24 h and 48 h represent the four inoculation time points; 1, 2 and 3 represent three independent biological replicates.

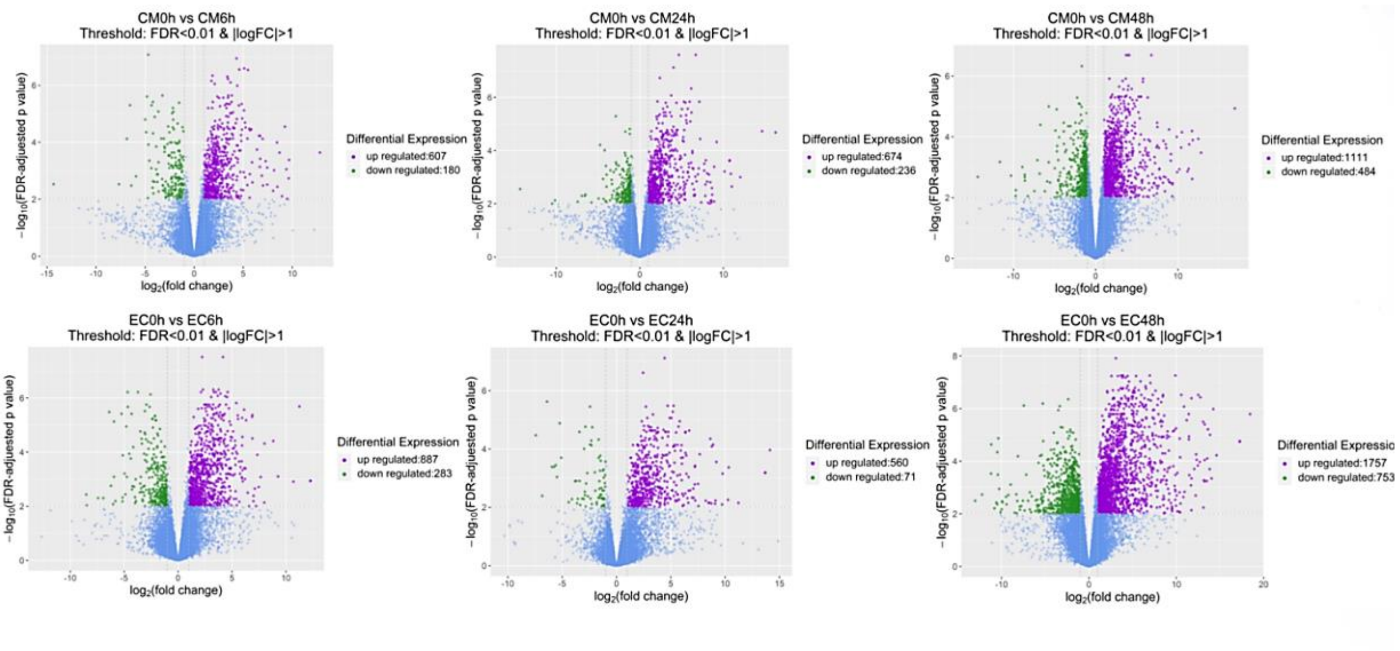

Figure S1 Volcano plots of differentially expressed genes (DEGs) in pepper's response to *P. capsici* infection

The purple dots are significantly up-regulated genes, the green dots are significantly down-regulated genes, and the blue dots are genes without significant changes.

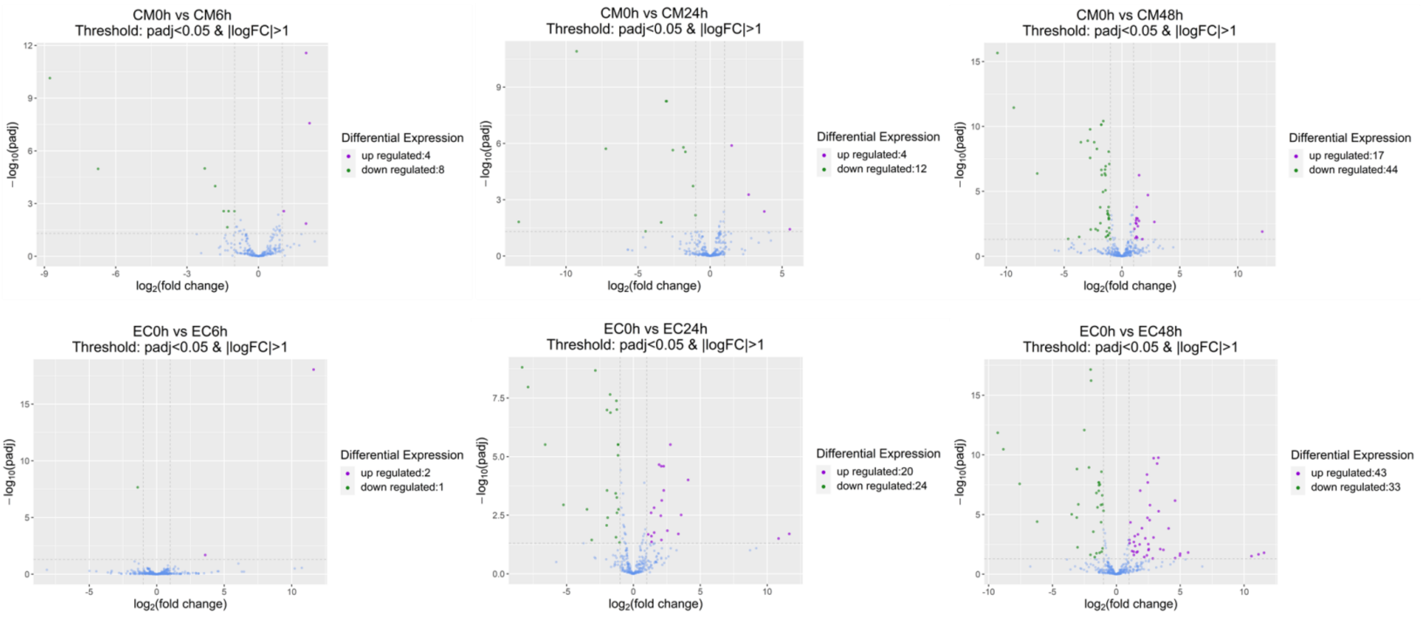

Figure S2 Volcano plots of differentially expressed miRNAs (DEMs) in pepper's response to *P. capsici* infection

The purple dots are significantly up-regulated DEMs, the green dots are significantly down-regulated DEMs, and the blue dots are DEMs without significant changes.
